# African Genetic Ancestry, Structural and Social Determinants of Health, and Mortality in Black Adults

**DOI:** 10.1001/jamanetworkopen.2025.10016

**Published:** 2025-05-13

**Authors:** Hari S. Iyer, Iona Cheng, Chidinma Opara, Katherine Lin, Nur Zeinomar, Loïc Le Marchand, Lynne Wilkens, Salma Shariff-Marco, David V. Conti, Christopher A. Haiman, Scarlett L. Gomez, Timothy R. Rebbeck

**Affiliations:** 1 Section of Cancer Epidemiology and Health Outcomes, Rutgers Cancer Institute, New Brunswick, New Jersey; 2 Department of Epidemiology and Biostatistics, University of California, San Francisco; 3 Epidemiology Program, University of Hawaii Cancer Center, Honolulu; 4 Center for Genetic Epidemiology, Department of Population and Public Health Sciences, Keck School of Medicine, University of Southern California, Los Angeles; 5 Division of Population Sciences, Dana-Farber Cancer Institute, Boston, Massachusetts; 6 Department of Epidemiology, Harvard T.H. Chan School of Public Health, Boston, Massachusetts

## Abstract

**Importance:**

Although structural and social determinants of health (SSDH) have been consistently associated with health disparities, percentage African genetic ancestry (AGA) has been suggested as a risk factor associated with common diseases in Black populations. Appropriate use and interpretation of percentage AGA in understanding health disparities has been complicated by the fact that percentage AGA is correlated with genetic and nongenetic factors.

**Objective:**

To evaluate associations of SSDH with mortality in the context of percentage AGA and how percentage AGA is correlated with SSDH.

**Design, Setting, and Participants:**

This cohort study investigated data from the Multiethnic Cohort (MEC) Study, in which participants were enrolled from 1993 through 1996 and followed up until death or censoring on December 31, 2019. Participant data were analyzed between March and June 2023. The population-based sample was predominantly from Los Angeles County, California, consisting of self-identified Black adults aged 45 to 75 years who enrolled into the MEC Study; completed a baseline demographic, clinical, and lifestyle questionnaire; and provided biospecimens.

**Exposures:**

The Index of Concentration at the Extremes (ICE), capturing social polarization based on income and racial composition, and a neighborhood socioeconomic status (NSES) index were computed from the 1990 Census, scaled to county-specific quintiles, and linked to residential census tracts at study enrollment. Percentage AGA was estimated using 21 431 single-nucleotide variations based on similarity with African continental referent data.

**Main Outcomes and Measures:**

Multivariable hazard ratios (HRs) for all-cause mortality were estimated from Cox models. Correlation of percentage AGA with SSDH measures was described.

**Results:**

After exclusions, 9685 participants were included (mean [SD] age, 61.0 [8.9] years; 5593 female [57.7%]), with a mean (SD) percentage AGA of 75.0% (14.0%). There were 5504 deaths over 204 463 person-years of follow-up. Comparing the most with least advantaged quintile, income ICE (adjusted HR [aHR], 1.30; 95% CI, 1.16-1.45) and NSES (aHR, 1.37, 95% CI, 1.20-1.56) were associated with lower all-cause mortality. Minimal changes were observed after adjusting for percentage AGA; for example, comparing the most with least advantaged quintile, NSES (aHR, 1.36; 95% CI, 1.19-1.55) remained associated with lower all-cause mortality. There was no association between percentage AGA and mortality after adjustment (aHR per 10–percentage point change in percentage AGA, 1.01; 95% CI, 0.99-1.03).

**Conclusions and Relevance:**

In this study, associations of SSDH with mortality persisted with adjustment for percentage AGA. Findings support the hypothesis that SSDH should be the primary factors to consider for eliminating health disparities.

## Key Points

**Question:**

In studies of structural and social determinants of health (SSDH) and mortality in Black adults, is percentage African genetic ancestry (AGA) independently associated with mortality?

**Findings:**

In this cohort study of 9685 Black adults from Los Angeles County, higher Index of Concentration at the Extremes measures and neighborhood socioeconomic status were associated with lower mortality but including percentage AGA did not change results. In contrast, percentage AGA was not associated with mortality after adjustment for behavioral, sociodemographic, and SSDH factors, and including percentage AGA did not improve model fits.

**Meaning:**

In this study, SSDH was associated with mortality independently of percentage AGA, and SSDH but not AGA was correlated with mortality in Black adults.

## Teaser

This cohort study investigates the independent associations of African genetic ancestry and structural and social determinants of health with mortality among Black US adults.

## Introduction

Prevailing frameworks aimed at understanding racial health disparities posit that excess mortality for Black US residents compared with other racial groups could be driven by multilevel (individual to societal) factors involving genetic and nongenetic causes.^1^ Structural racism, or the normalization of political systems that create privilege for dominant racial groups while disadvantaging others,^2^ may contribute to fewer educational and economic opportunities, barriers to healthy lifestyles, and higher exposure to environmental harms for Black populations.^3,4,5^ Neighborhood structural and social determinants of health (SSDH) include racial residential segregation and lower socioeconomic status (SES)^6^ and are often correlated with poorer access to care.^7,8^

In addition, genetic admixture and polygenic disease risk scores within self-identified racial and ethnic (SIRE) groups, based on single-nucleotide variants (formerly, single-nucleotide polymorphisms), have been associated with higher risk of cardiovascular disease and cancers,^9,10,11,12,13,14,15^ the leading causes of death in Black adults.^16^ Within Black populations, the frequencies of certain known genetic risk variants for prostate and breast cancer incidence are higher among individuals who are genetically similar to native West African populations.^10,11,12,14,17^ Risk variants with ancestrally differentiated variant frequencies are found across many health conditions and population groups. Available evidence suggests relatively stronger associations of SSDH with mortality disparities between Black and White adults compared with genetic factors.^18,19,20,21,22^ However, some argue that studying clusters of genetic similarity based on aggregated risk allele frequencies may reveal distinct etiologic pathways that could be exploited through targeted early detection or treatment for certain SIRE-defined groups.^23,24^

Genetic ancestry (GA) has been proposed for classifying ancestrally similar populations.^25^ Unlike polygenic risk scores, which are constructed with disease prediction in mind, GA measures were designed to understand population stratification. GA measures estimate genomic relatedness between individuals by comparing a large set of genetic markers to establish reference populations using cluster-based statistical methods.^26^ By clustering individuals based on the similarity of their genomes to those of reference populations, this approach can reveal evolutionary, geographic, and historical processes that explain modern-day human genetic variation.^27^

However, use and interpretation of GA in health disparities research requires careful scrutiny (eBox in Supplement 1). Scientific journal editors and consensus panels have recently provided guidance for the use of GA measures in health disparities research.^28,29,30,31,32^ GA labels are based on SIRE, nationality, or other sociopolitical categorizations (eg, continental boundaries or shared cultural belonging).^33^ If measurement of GA relies on referent population labels that are sociopolitically determined and those sociopolitical factors are correlated with health outcomes, GA measures may be inextricably linked to SSDH and other sociocultural factors that result in health disparities.^28,29^ Health researchers studying genetic admixture must therefore carefully assess how GA is used and interpreted, particularly when referent labels are based on SIRE or nationality, to avoid perpetuating incorrect and harmful claims of racial essentialism.^29,34,35^

To illustrate conceptual and interpretational challenges that may arise when using percentage African GA (AGA) in health disparities research, we evaluated whether (1) associations between SSDH and mortality differed in models with vs without adjustment for percentage AGA and (2) percentage AGA was statistically correlated with SSDH and behavioral factors among self-identified Black or African American participants from the Multiethnic Cohort (MEC).^36^ We hypothesized that percentage AGA would not be associated with mortality after SSDH were considered, implying that associations of percentage AGA with mortality are due to unmeasured confounding by SSDH.

## Methods

### Study Population and Design

This cohort study was approved by Institutional Review Boards (IRBs) of the Dana-Farber Cancer Institute; University of California, San Francisco; University of Southern California; and University of Hawai‘i. All IRBs waived the informed consent requirement and Health Insurance Portability and Accountability Act of 1996 authorization because the study presented minimal risk, involving no patient contact, and because contacting all individuals would be prohibitively costly. We followed the Strengthening the Reporting of Observational Studies in Epidemiology (STROBE) reporting guideline.

The MEC is an ongoing prospective cohort study of 5 major SIRE groups recruited from Hawai‘i and California.^36^ We obtained data from 34 921 self-identified Black participants who were recruited by targeting census tracts with 65% or greater proportions of Black residents in Los Angeles County according to census classifications. In addition, Health Care Financing Administration files were used to recruit Black adults aged 65 years and older at study entry. Participants aged 45 to 75 years completed questionnaires at baseline (1993-1996), in which they reported information about demographic characteristics, lifestyles, and comorbidities. Blood and buccal samples were collected starting in 1995 for nested case-control studies of common cancers and genomic analyses from a subsample of consenting participants. Follow-up occurred until death or censorship on December 31, 2019, whichever came first. Deaths were ascertained through linkage to California death files and the National Death Index, with a median (range) follow-up of 23.9 (1.8-26.7) years. Residential addresses were geocoded at baseline to parcel or street centerline data. Geocodes were used to assign neighborhood data at the census tract level. Details regarding design and population characteristics of the MEC have been described previously.^36,37^

We restricted the eligible study population to 11 073 self-identified Black or African American (hereafter, *Black*) participants with available genome-wide single nucleotide variation data. We excluded 34 participants outside of Los Angeles County to reduce potential confounding due to geographic differences, 274 participants with missing geocoded residential addresses, and 48 participants with missing covariate data. Because we sought to examine risk patterns in Black individuals, whose history and cultural context differ from that of recent immigrants from the African continent, we further excluded 305 participants whose parents did not self-identify as Black and 725 individuals who were foreign-born, for a final sample size of 9685 participants.

### Structural and Social Determinants of Health

We considered neighborhood segregation by race and income as a proxy for neighborhood-level structural racism,^38^ operating distinctly from neighborhood SES (NSES), which captures economic and occupational hierarchies and material resources and which we conceptualize as a consequence of structural racism.^3,39^ We used the Index of Concentration at the Extremes (ICE), a segregation measure of “social spatial polarization.”^40^ The ICE is a fraction, where the numerator is the difference between the total number of people occupying an advantaged vs disadvantaged position in society with respect to race and income and the denominator is the total number of people in the census tract. ICE values range from −1 to 1, with values closer to −1 indicating concentration of disadvantage, while values closer to 1 indicate concentration of privilege.

Data to compute the ICE were obtained from the 1990 decennial Census at tract level. We calculated an income ICE measure based on lowest (20th percentile) vs highest (80th percentile) quintile of household income and a racialized income ICE measure, which classified census tracts based on concentration of non-Hispanic Black residents in the lowest quintile of income vs non-Hispanic White residents in the highest quintile of income.^40^ We also calculated a race ICE measure based on the concentration of non-Hispanic Black to White residents in each census tract. Because of limited variability in race ICE in this study population given the sampling strategy, income ICE and racialized income ICE measures were selected as primary structural measures.

A composite NSES score was developed using data obtained from the 1990 decennial Census at census tract level as part of the California Neighborhoods Data System geospatial database.^41^ Principal component analysis was used to produce a summary index incorporating the following measures: median income, percentage population below 200% of the poverty level, median rent, median home value, proportion with a blue collar job,^42^ proportion older than 16 years in the workforce without a job, and a previously defined education index that weights populations in each census tract by the years required to obtain each level of education.^43^ Further details regarding the methodology used to compute the NSES index are available elsewhere.^42^

### African Genetic Ancestry

MEC participants have previously been genotyped on a variety of Illumina arrays, with a density ranging from 500 000 to 2.5 million variants.^44^ Following the same quality control pipeline and imputation procedures, all array data have been imputed via the Michigan Imputation Server using the 1000 Genome Projects phase 3 panel and the PAGE Global Reference Panel.^45^ Full descriptors of reference populations and countries of origin used to label AGA clusters in the MEC are provided in the eMethods and eTable 1 in Supplement 1.

We estimated the percentage AGA using the Admixture program^46^ in all MEC participants with array data using 21 431 common variants not in linkage disequilibrium (*R*^2^ <0.10 and minor allele frequency >1% in all racial and ethnic populations) genotyped across all arrays. The number of clusters estimated in the entire MEC sample was limited to *K* = 5. These clusters correlated with continental ancestry groupings as represented by reference panel labels, including European, African, and East Asian, with additional clusters of suspected Amerindian ancestry within the MEC Latino racial and ethnic group and Polynesian ancestry identified in the MEC Native Hawaiian racial and ethnic group. Here, because individuals involved in the analysis were restricted to self-identified Black participants, the percentage AGA used was the cluster labeled as African ancestry.

### Statistical Analysis

We examined Spearman correlations between percentage AGA and SSDH. We included the following measures as covariates in subsequent analyses based on prior work examining associations of percentage AGA, NSES, and mortality in the MEC and other cohorts, which were informed by directed acyclic graphs^19,20,37^: 10-year birth cohort to capture generational influences and because age was included as a time scale in survival models (categorical age groups: 45 to 51.7 years, 51.7 to 58.4 years, 58.4 to 65.2 years, 65.2 to 69.8 years, and 69.8 to 78.0 years), sex (male and female), baseline measure of smoking status (categorical groups: current, former, and never), marital status (categorical groups: married, separated, divorced, widowed, never married, and unknown), educational attainment (<high school, ≥high school, and unknown), body mass index (BMI; calculated as weight in kilograms divided by height in meters squared) and BMI at age 21 years (categorical groups for both: <18.5, 18.5 to <25.0, 25 to <30, and ≥30), history of comorbidities (hypertension, diabetes, cardiovascular disease, cancer, all yes, and no), physical activity in Metabolic Equivalent Task-hours per week (quintiles), and Alternative Healthy Eating Index (AHEI) diet scores (quintiles). Given that NSES and covariates were assessed at baseline, covariates were considered as confounders rather than mediators.

Median age at death was estimated from unadjusted Kaplan-Maier survival curves. Cox proportional hazards models were fitted using age as the time scale to estimate hazard ratios (HRs) for all-cause mortality and 95% CIs. We fit models using linear terms scaling to a 10–percentage point increase for percentage AGA or IQR for NSES and ICE measures. To evaluate the potential for policy-relevant thresholds, we also modeled exposures using quintiles (reverse coded) and an ordinal test for trend. To facilitate comparisons with other population-based studies in the same study setting, we applied quintile cut points for census tracts in Los Angeles County rather than applying study-specific quintile cut points. All models were fit using robust sandwich errors to account for census tract–level clustering.^47^

We separately estimated associations of NSES and ICE measures with mortality by sequentially adjusting for the following variables taken at time of study enrollment. Model 1 (minimally adjusted) was adjusted for age and sex. Model 2 (confounding) was additionally adjusted for smoking, marital status, educational attainment, BMI (at the time of study entry), BMI at age 21 years, history of comorbidities, physical activity, and AHEI. Model 3 included all factors in model 2 plus percentage AGA. We did not analyze NSES and ICE measures jointly due to collinearity.

We compared model performance from confounding-adjusted Cox models that separately included percentage AGA, NSES, and ICE measures. Likelihood ratio tests were used to estimate *P* values for resulting improvement in model fit. We also calculated Akaike information criterion, Bayes information criterion, and the concordance statistic (an analog of the *C* statistic that accounts for censoring in time-to-event analyses^48^).

All analyses were performed using R statistical software version 4.2.2 (R Project for Statistical Computing). All hypothesis tests were 2-sided, with α = .05. Data were analyzed from March to June 2023. Further details are provided in the eMethods in Supplement 1.

## Results

### Participant Characteristics

Among 9685 eligible participants (mean [SD] age, 61.0 [8.9] years; 5593 female [57.7%]), the mean (SD) percentage AGA was 75.0% (4.0%). Frequency distributions of multilevel factors among Black participants in the MEC stratified by NSES are reported in Table 1. Among 424 MEC participants in NSES quintile 1 (least deprived) compared with 2890 participants in quintile 5 (most deprived), those in quintile 1 were more likely to have a high school education or more (360 participants [84.9%] vs 1487 participants [51.5%]), be married (286 participants [67.5%] vs 1194 participants [41.3%]), and be in the highest quintile of moderate to vigorous physical activity (114 participants [26.9%] vs 491 participants [17.0%]).

**Table 1.** Study Participant Characteristics

Characteristic	Participants, No. (%)^a^					Overall (N = 9685)	*P* value^c^
	NSES Q1 (least deprived) (n = 424)^b^	NSES Q2 (n = 1514)^b^	NSES Q3 (n = 1718)^b^	NSES Q4 (n = 3139)^b^	NSES Q5 (most deprived) (n = 2890)^b^		
NSES Index, range	0.93 to 2.55	0.29 to 0.92	−0.35 to 0.28	−1.13 to −0.34	−3.63 to −1.12		
AGA, mean (SD), %	70.02 (15.50)	72.12 (15.23)	74.38 (13.64)	75.14 (13.96)	77.68 (12.80)	75.07 (14.01)	<.001
Age, mean (SD), y	64.76 (8.23)	60.16 (8.98)	61.28 (8.99)	61.30 (8.64)	60.50 (8.84)	61.03 (8.85)	<.001
Sex^d^							
Female	209 (49.3)	827 (54.6)	1016 (59.1)	1789 (57.0)	1752 (60.6)	5593 (57.7)	<.001
Male	215 (50.7)	687 (45.4)	702 (40.9)	1350 (43.0)	1138 (39.4)	4092 (42.3)	
Educational attainment							
<High school	58 (13.7)	275 (18.2)	473 (27.5)	1142 (36.4)	1368 (47.3)	3316 (34.2)	<.001
≥High school	360 (84.9)	1223 (80.8)	1226 (71.4)	1966 (62.6)	1487 (51.5)	6262 (64.7)	
Unknown	6 (1.4)	16 (1.1)	19 (1.1)	31 (1.0)	35 (1.2)	107 (1.1)	
Marital status							
Married	286 (67.5)	962 (63.5)	895 (52.1)	1602 (51.0)	1194 (41.3)	4939 (51.0)	<.001
Separated	12 (2.8)	44 (2.9)	80 (4.7)	132 (4.2)	232 (8.0)	500 (5.2)	
Divorced	72 (17.0)	259 (17.1)	424 (24.7)	685 (21.8)	721 (24.9)	2161 (22.3)	
Widowed	33 (7.8)	172 (11.4)	223 (13.0)	481 (15.3)	482 (16.7)	1391 (14.4)	
Never married	14 (3.3)	69 (4.6)	77 (4.5)	203 (6.5)	229 (7.9)	592 (6.1)	
Unknown	7 (1.7)	8 (0.5)	19 (1.1)	36 (1.1)	32 (1.1)	102 (1.1)	
AHEI 2010, quintile							
First (30.8 to 57.0)	65 (15.3)	294 (19.4)	342 (19.9)	634 (20.2)	635 (22.0)	1970 (20.3)	<.001
Second (57.1 to 62.7)	91 (21.5)	301 (19.9)	354 (20.6)	599 (19.1)	589 (20.4)	1934 (20.0)	
Third (62.8 to 66.6)	76 (17.9)	259 (17.1)	326 (19.0)	632 (20.1)	632 (21.9)	1925 (19.9)	
Fourth (66.7 to 77.2)	85 (20.0)	336 (22.2)	342 (19.9)	636 (20.3)	550 (19.0)	1949 (20.1)	
Fifth (77.3 to 97.8)	107 (25.2)	324 (21.4)	354 (20.6)	638 (20.3)	484 (16.7)	1907 (19.7)	
Smoking status							
Never	187 (44.1)	628 (41.5)	612 (35.6)	1125 (35.8)	1047 (36.2)	3599 (37.2)	<.001
Former	190 (44.8)	654 (43.2)	755 (43.9)	1303 (41.5)	1104 (38.2)	4006 (41.4)	
Current	43 (10.1)	217 (14.3)	328 (19.1)	682 (21.7)	704 (24.4)	1974 (20.4)	
Missing	4 (0.9)	15 (1.0)	23 (1.3)	29 (0.9)	35 (1.2)	106 (1.1)	
BMI							
Mean (SD)	26.79 (4.24)	27.50 (4.63)	28.47 (5.22)	28.63 (5.67)	29.51 (5.91)	28.60 (5.51)	<.001
At age 21 y, mean (SD)	21.65 (3.10)	21.77 (3.03)	21.90 (3.11)	22.04 (3.48)	22.35 (3.86)	22.04 (3.46)	<.001
MET-h/wk (moderate + vigorous) quintile							
First (0 to 1.2)	66 (15.6)	273 (18.0)	333 (19.4)	645 (20.5)	671 (23.2)	1988 (20.5)	<.001
Second (1.3 to 2.2)	75 (17.7)	322 (21.3)	366 (21.3)	715 (22.8)	685 (23.7)	2163 (22.3)	
Third (2.3 to 4.0)	86 (20.3)	298 (19.7)	369 (21.5)	554 (17.6)	530 (18.3)	1837 (19.0)	
Fourth (4.1 to 6.6)	83 (19.6)	304 (20.1)	310 (18.0)	604 (19.2)	513 (17.8)	1814 (18.7)	
Fifth (6.7 to 81.5)	114 (26.9)	317 (20.9)	340 (19.8)	621 (19.8)	491 (17.0)	1883 (19.4)	
High blood pressure	227 (53.5)	707 (46.7)	922 (53.7)	1689 (53.8)	1603 (55.5)	5148 (53.2)	<.001
History of diabetes	176 (10.2)	244 (12.2)	278 (13.7)	280 (14.0)	315 (16.2)	1293 (13.4)	<.001
History of CVD	202 (11.8)	247 (12.4)	227 (11.2)	272 (13.6)	256 (13.2)	1204 (12.4)	.14
History of cancer	207 (12.0)	207 (10.4)	200 (9.9)	209 (10.4)	225 (11.6)	1048 (10.8)	.17
Income ICE, quintile^e^							
First (0.15 to 1.00) [most privileged]	248 (58.5)	537 (35.5)	0	0	0	785 (8.1)	<.001
Second (−0.02 to 0.14)	168 (39.6)	840 (55.5)	378 (22.0)	20 (0.6)	0	1406 (14.5)	
Third (−0.11 to −0.01)	8 (1.9)	121 (8.0)	785 (45.7)	252 (8.0)	1 (<0.1)	1167 (12.0)	
Fourth (−0.27 to −0.12)	0	16 (1.1)	530 (30.8)	1704 (54.3)	160 (5.5)	2410 (24.9)	
Fifth (−1.00 to −0.26) [most deprived]	0	0	25 (1.5)	1163 (37.1)	2729 (94.4)	3917 (40.4)	
Race ICE, quintile^e^^,^^f^							
First (0.77 to 1.00) [most privileged]	92 (21.7)	43 (2.8)	31 (1.8)	27 (0.9)	0	193 (2.0)	<.001
Second (0.55 to 0.76)	63 (14.9)	133 (8.8)	90 (5.2)	61 (1.9)	0	347 (3.6)	
Third (0.24 to 0.54)	72 (17.0)	99 (6.5)	190 (11.1)	83 (2.6)	27 (0.9)	471 (4.9)	
Fourth (0.03 to 0.23)	28 (6.6)	27 (1.8)	55 (3.2)	106 (3.4)	54 (1.9)	270 (2.8)	
Fifth (−1.00 to 0.02) [most deprived]	169 (39.9)	1212 (80.1)	1352 (78.7)	2862 (91.2)	2809 (97.2)	8404 (86.8)	
Racialized income ICE quintile^e^^,^^f^							
First (0.21 to 1.00) [most privileged]	182 (42.9)	27 (1.8)	0	0	0	209 (2.2)	<.001
Second (0.09 to 0.20)	86 (20.3)	188 (12.4)	69 (4.0)	7 (0.2)	0	350 (3.6)	
Third (0.04 to 0.09)	33 (7.8)	124 (8.2)	227 (13.2)	88 (2.8)	2 (0.1)	474 (4.9)	
Fourth (−0.02 to 0.03)	0	229 (15.1)	103 (6.0)	138 (4.4)	35 (1.2)	505 (5.2)	
Fifth (−1.00 to −0.01) [most deprived]	123 (29.0)	946 (62.5)	1319 (76.8)	2906 (92.6)	2853 (98.7)	8147 (84.1)	

Abbreviations: AGA, African genetic ancestry; AHEI, Alternative Healthy Eating Index; BMI, body mass index (calculated as weight in kilograms divided by height in meters squared); CVD, cardiovascular disease; ICE, Index of Concentrations at the Extremes; MET, metabolic equivalent task; NSES, neighborhood socioeconomic status; Q, quintile.

^a^ Cell percentages may not add up to 100% due to rounding. All characteristics were assessed at baseline unless otherwise specified. NSES and ICE measures were reverse coded.

^b^ Los Angeles County–specific census tract cut points were applied, so quintiles are not of equal size.

^c^ The χ^2^ test for independence was used for categorical variables, and the independent 2-sample *t* test was used for means.

^d^ This study was conducted at a time when nonbinary presentation and disclosure was less common, and so binary gender (male and female) was the only gender categorization available in this study.

^e^ Due to high collinearity and sparse cells when jointly stratifying for NSES and ICE measures, these measures were analyzed separately in regression models.

^f^ For race ICE and racialized income ICE, comparison is between the proportion non-Hispanic White and non-Hispanic Black in the census tract.

**Figure 1.**
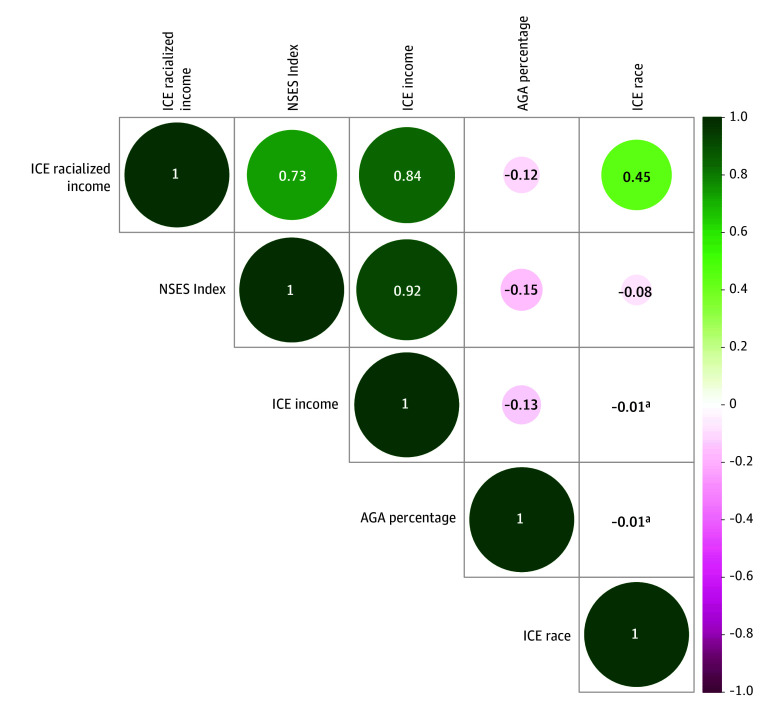
Spearman Correlations of Percentage African Genetic Ancestry (AGA) With Geospatial Social and Structural Determinants of Health Green indicates positive correlation; ICE, Index of Concentration at the Extremes; magenta, negative correlation; NSES, neighborhood socioeconomic status. ^a^Not statistically significant at 2-sided α = .05.

Spearman correlations of percentage AGA with census tract NSES and ICE measures ranged from −0.01 to −0.12 (Figure 1). Percentage AGA was correlated with NSES (*r* = −0.15; *P* < .001), ICE income (*r* = −0.13; *P* < .001), and racialized income ICE (*r* = −0.12; *P* < .001). Spearman correlations with other component census-tract SSDH are included in eFigure 1 in Supplement 1.

### Associations of SSDH and Percentage AGA With Mortality

There were 5504 all-cause deaths over 204 463 person-years of follow-up. Unadjusted Kaplan-Meier survival curves for all-cause mortality were estimated across quintiles of percentage AGA, NSES, and ICE measures (eFigure 2 in Supplement 1). There was no association between percentage AGA and age at death. Median age at death was lower comparing quintile 5 (most concentrated in disadvantage) with quintile 1 (most concentrated in advantage) for income ICE (85.3 years; 95% CI, 84.8-85.9 years vs 89.1 years; 95% CI; 88.2-90.3 years; *P* < .001), racialized income ICE (86.1 years; 95% CI, 85.8-86.5 years vs 90.1 years; 95% CI, 87.8-91.5 years; *P* < .001), and NSES (85.0 years; 95% CI, 84.5-85.6 years vs 89.5 years; 95% CI, 88.2, 90.9 years; *P* < .001).

**Figure 2.**
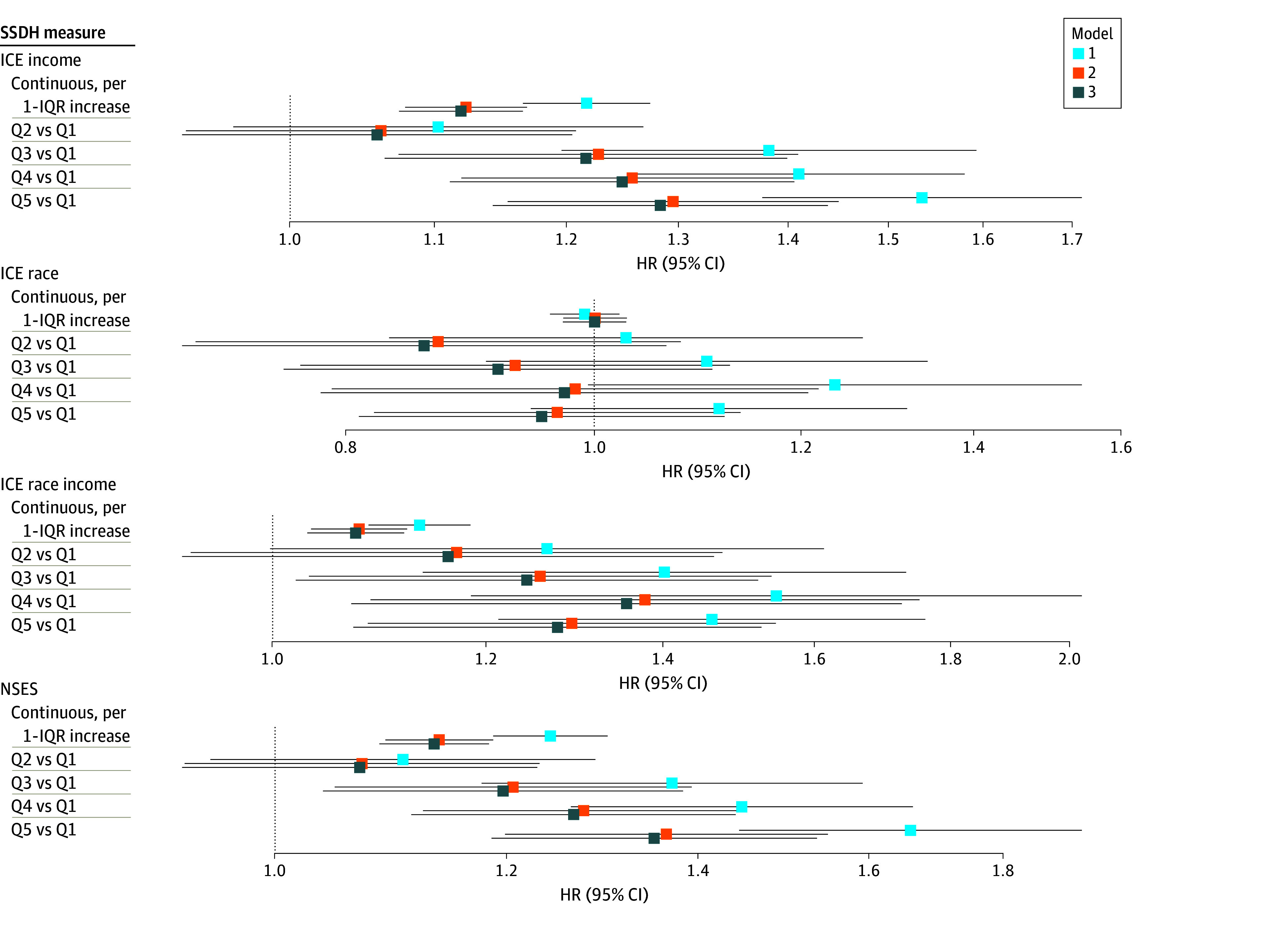
Associations of Measures of Neighborhood Structural and Social Determinants of Health (SSDH) With All-Cause Mortality Model 1 (minimally adjusted) was adjusted for birth cohort and sex. Model 2 (confounding) was adjusted for model 1 factors plus smoking status, marital status, educational attainment, body mass index (calculated as weight in kilograms divided by height in meters squared), body mass index at age 21 years, history of hypertension, history of diabetes, history of cardiovascular disease, history of cancer, physical activity, and Alternative Healthy Eating Index score. Model 3 was adjusted for all factors in model 2 plus percentage African Genetic Ancestry (AGA). Continuous factors were scaled to the IQR. See eTable 2 in Supplement 1 for hazard ratio (HR) and 95% CI tabular data. ICE indicates Index of Concentration at the Extremes; NSES, neighborhood socioeconomic status; Q, quintile.

**Figure 3.**
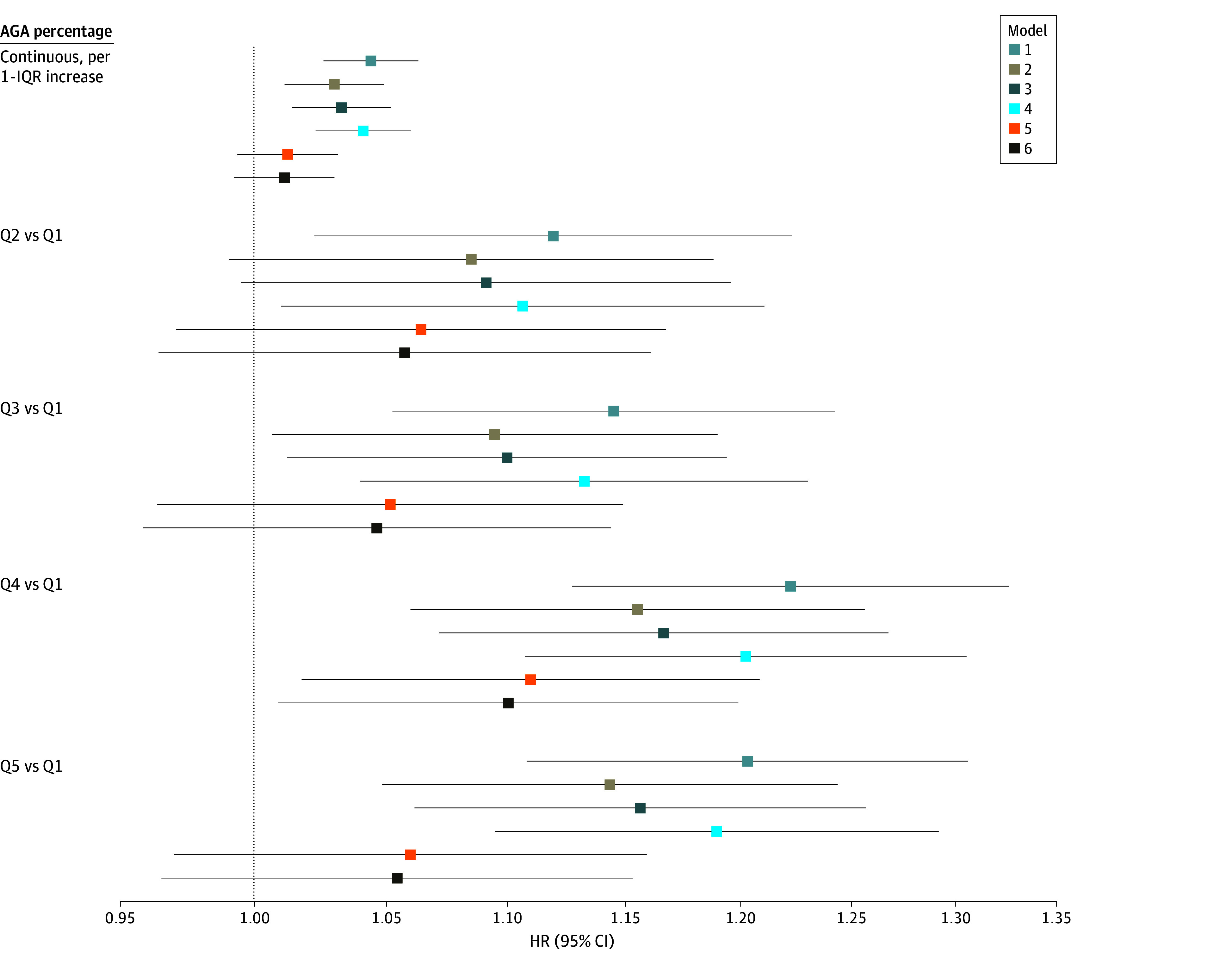
Associations Between Percentage African Genetic Ancestry (AGA) and All-Cause Mortality Model 1 (minimally adjusted) was adjusted for birth cohort and sex. Models 2, 3, and 4 were adjusted for model 1 factors plus neighborhood socioeconomic status, Index of Concentration at the Extremes income, and Index of Concentration at the Extremes racialized income, respectively. Model 5 (confounding) was adjusted for model 1 factors plus smoking status, marital status, educational attainment, body mass index (calculated as weight in kilograms divided by height in meters squared), body mass index at age 21 years, history of hypertension, history of diabetes, history of cardiovascular disease, history of cancer, physical activity, and Alternative Healthy Eating Index score. Model 6 was adjusted for model 5 factors plus racialized income Index of Concentration at the Extremes. Continuous factors were scaled to 10% AGA increase in African admixture vs European. See eTable 3 in Supplement 1 for hazard ratio (HR) and 95% CI tabular data. Q indicates quintile.

In fully adjusted Cox models for associations of SSDH with mortality (Figure 2; eTable 2 in Supplement 1) comparing quintile 5 with quintile 1, income ICE (adjusted HR [aHR], 1.29; 95% CI, 1.15-1.44; *P* for trend < .001) and NSES (aHR, 1.36; 95% CI, 1.19-1.55; *P* for trend < .001) were associated with higher all-cause mortality, but there was no association of race ICE with all-cause mortality (aHR, 1.00; 95% CI, 0.97-1.03; *P* for trend = .38). For racialized income ICE, quintile 5 was associated with higher all-cause mortality compared with quintile 1 (aHR, 1.28; 95% CI, 1.08-1.53), but the test for linear trend did not reach statistical significance (*P* for trend = .06). Adjusting for behavioral and demographic factors led to the sharpest attenuation of effect estimates, with limited impact of further adjustment for percentage AGA. For example, in the minimally adjusted model, comparing quintile 5 with quintile 1 of income ICE, the HR was 1.54 (95% CI, 1.38-1.72). Adjusting for confounding attenuated this estimate to 1.30 (95% CI, 1.16-1.45), which was similar to the model with additional adjustment for percentage AGA. Comparing quintile 5 with quintile 1 of NSES, the HR was 1.67 (95% CI, 1.45-1.91) in the minimally adjusted model. After adjusting for confounding, the aHR was 1.37 (95% CI, 1.20-1.56), which was similar to the model after further adjustment for percentage AGA. Unlike SSDH, percentage AGA had no association with all-cause mortality in fully adjusted models (aHR per 10% increase in African admixture, 1.01; 95% CI, 0.99-1.03) (Figure 3; eTable 3 in Supplement 1).

Including SSDH in models with behavioral and demographic factors led to superior model fit statistics (Table 2). Models including NSES and income ICE modeled as linear terms or as quintiles had significantly better model fit (likelihood ratio test *P* < .001 for all comparisons with base models). Models with continuous income ICE had the lowest Akaike information criterion (75 603.13) and Bayes information criterion (75 814.75). There was limited difference in concordance statistics calculated with each set of factors. Including percentage AGA did not improve model fit.

**Table 2.** Improvement in Fit After Inclusion of SSDH vs Percentage AGA

Exposure	Likelihood ratio test *P* value vs base model^a^	Akaike information criterion	Bayes information criterion	Concordance statistic
Base model^b^	NA	75 627.16	75 832.17	0.6474
**SSDH**				
NSES				
Continuous^c^	<.001	75 603.25	75 814.87	0.6487
Quintile	<.001	75 603.97	75 835.43	0.6490
Income ICE				
Continuous^c^	<.001	75 603.13	75 814.75	0.6489
Quintile	<.001	75 607.76	75 839.22	0.6489
Race ICE				
Continuous^c^	.83	75 629.12	75 840.74	0.6474
Quintile	.64	75 632.57	75 864.03	0.6473
Racialized income ICE				
Continuous^c^	.001	75 618.83	75 830.45	0.6480
Quintile	.051	75 625.70	75 857.17	0.6478
**Percentage AGA**				
Continuous^d^	.21	75 627.60	75 839.23	0.6476
Quintile	.23	75 629.54	75 861.00	0.6479

Abbreviations: AGA, African genetic ancestry; ICE, Index of Concentration at Extremes; NA, not applicable; NSES, neighborhood socioeconomic status; SSDH, social determinants of health.

^a^ Improvement in fit is shown for Cox models for all-cause mortality.

^b^ All models were adjusted for clustering at the census tract level using robust sandwich errors. Models were sequentially adjusted for birth cohort, sex, smoking status, marital status, educational attainment, body mass index (calculated as weight in kilograms divided by height in meters squared), body mass index at age 21 years, history of hypertension, history of diabetes, history of cardiovascular disease, history of cancer, physical activity, and Alternative Healthy Eating Index score.

^c^ Scaled to IQR for socioeconomic variables.

^d^ Scaled to 10% increase in percentage AGA.

## Discussion

In this cohort study of self-identified Black adults, higher neighborhood deprivation was associated with higher all-cause mortality, and adjustment for percentage AGA did not change associations of census tract–level SSDH with mortality in Black adults. There was no robust association of percentage AGA with mortality. These results suggest that although percentage AGA was associated with mortality for Black adults before accounting for SSDH, this association was no longer present after this adjustment. Moreover, because percentage AGA was modestly correlated with measured SSDH, any residual correlation between percentage AGA and health may reflect unmeasured correlates of SSDH (eg, housing and health care access).^19,29^

For most common diseases, social and environmental factors contribute more strongly to excess mortality than genetic variation in Black compared with other SIRE groups.^18,19,20,21,22,49^ Our findings support earlier studies showing that failing to account for SSDH may overstate the strength of associations of percentage AGA with health-related end points.^18,19,20,21,29,50,51,52,53^ Adjusting for individual demographics, behavioral factors, and NSES attenuates associations between percentage AGA and health outcomes, ranging from overall mortality^19^ to breast cancer,^20,51^ lung cancer,^54,55^ hypertension,^21,50^ and cardiovascular disease.^52,56^ Together with these earlier studies, our findings suggest that for mortality, SSDH are more important drivers of health disparities in Black populations than percentage AGA. SSDH measures capture current and historical social processes that explain SIRE-based variation in environmental, behavioral, and access-related risk factors that influence health outcomes. Percentage AGA, as a genetic construct, should not substitute or replace social constructs like SIRE or SSDH when explaining health disparities.^29,53,57,58,59^

Our data support calls for thoughtful consideration of the choice of population descriptors.^28,29,30,31,32^ SIRE reflects how an individual socially identifies, while GA captures descent-related genetic similarity between individuals in a population with referent groups. Neighborhood deprivation and residential racial and economic segregation were associated with higher mortality in Black adults, reflecting adverse health effects of poor social conditions.^1,60^ Reported associations of NSES and ICE with mortality in Black adults may capture pathways involving health care access, educational and economic opportunities, and behavioral factors, which can serve as targets for narrowing health disparities.^6,39,57,61^

Our study also suggests that because social conditions play a major role in explaining SIRE-based health differences, GA may have limited use for advancing understanding of causes of racial disparities. GA captures descent-related genetic variation in populations, which cluster based on historical geographic, migration, and mixing patterns,^25,62,63^ but this variation does not reflect experiences of modern-day SIRE groups. Some high-risk genetic variants for disease cluster within SIRE groups, but these examples are rare, and use of risk variants themselves will offer superior risk prediction than GA.^62^ Therefore findings from these studies, although illuminating for disease etiology, would have limited impact on narrowing racial disparities in health.^22^ For studies where adverse social conditions are the main focus, polygenic risk scores for common diseases that are derived from diverse populations could be more useful than GA for risk stratification based on genetic susceptibility.^63^

### Strengths and Limitations

Despite several strengths, including a large population-based sample and adjustment for sociodemographic and behavioral factors with substantial follow-up time, this study also has some limitations. We did not account for all individual-level environmental exposures, medical interventions, or SSDH that may be associated with mortality outcomes, although we included many behavioral, demographic, and contextual covariates. Future studies should consider additional multilevel data, including time-varying behavioral measures and specific environmental attributes, to evaluate mediating pathways from SSDH and health across different racial groups to identify targets for interventions. Our results from Black adults in Los Angeles County may not be generalizable to other geographic settings or demographic groups. Our measures of SSDH were assessed at baseline but may have changed during the study period through residential mobility or gentrification. Because our goal was to illustrate conceptual challenges with examining AGA in association with excess mortality in Black adults, the potential use of GA-based genetic risk scores for cancer, cardiovascular disease, or other chronic diseases^56^ was beyond the scope of this study. Future studies should leverage genetic similarity measures that are developed without reference to existing sociopolitical groupings^29^ and rely purely on genetics for stratification (eBox in Supplement 1).

## Conclusions

In this cohort study of Black individuals, adverse SSDH were associated with higher mortality. These associations persisted after adjustment for percentage AGA. Measures capturing adverse social conditions rather than percentage AGA were more informative for understanding factors associated with mortality. These findings suggest that instead of percentage AGA, polygenic risk scores should be used to capture causal etiologies across populations. Furthermore, the purpose of using genomic or environmental measures in studies of racial disparities should be carefully considered and chosen based on clear etiological hypotheses.

## Notes

Supplement 1.
eMethods.
**eBox.** Challenges and Recommendations for Appropriate Use of Genetic Ancestry in Health Disparities Research **eTable 1.** Population Descriptors Used for Genetic Ancestry Clusters in the Multiethnic Cohort **eTable 2.** Hazard Ratios for Associations Between Measures of Neighborhood Structural and Social Determinants With All-Cause Mortality Among Self-Identified Black Adults From the Multiethnic Cohort **eTable 3.** Hazard Ratios for Associations Between Percentage African Genetic Ancestry and All-Cause Mortality Among Self-Identified Black Adults From the Multiethnic Cohort **eFigure 1.** Spearman Correlations for Geospatial Social and Structural Determinants of Health With Percentage African Genetic Ancestry **eFigure 2.** Kaplan-Meier Survival Curves for Associations of Percentage African Genetic Ancestry and Structural and Social Determinants of Health and All-Cause Mortality Among Self-Identified Black Participants From the Multiethnic Cohort Using Los Angeles County–Specific Cut Points

Supplement 2.
Data Sharing Statement
