# Supplementary material for: African Genetic Ancestry, Structural and Social Determinants of Health, and Mortality in Black Adults
Source: JAMA Netw Open. 2025 May 13;8(5):e2510016. doi: 10.1001/jamanetworkopen.2025.10016 (PMC12076178; doi:10.1001/jamanetworkopen.2025.10016)
Supplement: Supplement 1. — eMethods. eBox. Challenges and Recommendations for Appropriate Use of Genetic Ancestry in Health Disparities Research eTable 1. Population Descriptors Used for Genetic Ancestry Clusters in the Multiethnic Cohort eTable 2. Hazard Ratios for Associations Between Measures of Neighborhood Structural and Social Determinants With All-Cause Mortality Among Self-Identified Black Adults From the Multiethnic Cohort eTable 3. Hazard Ratios for Associations Between Percentage African Genetic Ancestry and All-Cause Mortality Among Self-Identified Black Adults From the Multiethnic Cohort eFigure 1. Spearman Correlations for Geospatial Social and Structural Determinants of Health With Percentage African Genetic Ancestry eFigure 2. Kaplan-Meier Survival Curves for Associations of Percentage African Genetic Ancestry and Structural and Social Determinants of Health and All-Cause Mortality Among Self-Identified Black Participants From the Multiethnic Cohort Using Los Angeles County–Specific Cut Points [file jamanetwopen-e2510016-s001.pdf]

Supplemental Online Content

Iyer HS, Cheng I, Opara C, et al. African genetic ancestry, structural and social determinants of health, and mortality in Black adults. *JAMA Netw Open*. 2025;8(5):e2510016.
doi:10.1001/jamanetworkopen.2025.10016

eMethods.

eBox 1. Challenges and Recommendations for Appropriate Use of Genetic Ancestry in Health Disparities Research

eTable 1. Population Descriptors Used for Genetic Ancestry Clusters in the Multiethnic Cohort

eTable 2. Hazard Ratios for Associations Between Measures of Neighborhood Structural and Social Determinants With All-Cause Mortality Among Self-Identified Black Adults From the Multiethnic Cohort

eTable 3. Hazard Ratios for Associations Between Percentage African Genetic Ancestry and All-Cause Mortality Among Self-Identified Black Adults From the Multiethnic Cohort

eFigure 1. Spearman Correlations for Geospatial Social and Structural Determinants of Health With Percentage African Genetic Ancestry

eFigure 2. Kaplan-Meier Survival Curves for Associations of Percentage African Genetic Ancestry and Structural and Social Determinants of Health and All-Cause Mortality Among Self-Identified Black Participants From the Multiethnic Cohort Using Los Angeles County–Specific Cut Points

This supplemental material has been provided by the authors to give readers additional information about their work.

© 2025 Iyer HS et al. *JAMA Network Open*.

eMethods

Genetic Ancestry reference labels

The MEC estimated percent African Genetic Ancestry (%AGA) with respect to 5 continental groupings (African, Indigenous American, East Asian, European, Polynesian). Population descriptors for African referent groups included: Esan in Nigeria, Gambian in Western Division – Mandinka, Luhya in Webuye, Kenya, Maasai in Kinyawa, Kenya, Mende in Serra Leone, and Yoruba in Ibadan, Nigeria. In addition, reference samples were derived from the Global Reference Panel developed as part of the Population Architecture using Genomics and Epidemiology (PAGE) study[30], which sampled individuals of indigenous origin from Puno, Peru (Quechuan/Aymaran ancestry), Easter Island, Chile (Rapa Nui), Oaxaca, Mexico, Honduras, Colombia, and the Northern Cape, South Africa (Nama, Khomani KhoeSan).

Estimating Associations of Social and Structural Determinants of Health with All-cause Mortality

We examined correlations between sociodemographic and lifestyle factors and our primary exposures (%AGA, nSES, ICE) using two sample independent t-tests and chi-squared tests of independence. We calculated crude Kaplan-Meier survival curves and log-rank tests for associations between each of these exposures and all-cause mortality. We fit models using linear terms scaled to an interquartile range (IQR) for nSES and ICE measures, and a 10% increase in African admixture for %AGA. To evaluate potential for policy-relevant thresholds, we also modeled exposures using quintiles and an ordinal test for trend. Los Angeles County-specific cutpoints were applied to compute quintiles for nSES and ICE measures.

Cox proportional hazards models were fitted using age as the time scale to estimate hazard ratios for mortality endpoints and 95% confidence intervals, sequentially adjusting for the following variables taken at time of study enrollment: Model 1 (Minimally adjusted): 10-year birth cohort (categorical age group: 45-51.7 years; 51.7-58.4 years, 58.4-65.2 years, 65.2-69.8 years, 69.8-78.0 years) and sex (male, female). Model 2 (Confounding): smoking status (categorical: current, former, never); marital status

© 2025 Iyer HS et al. *JAMA Network Open*.

(categorical: married, separated, divorced, widowed, never married, unknown), educational attainment (below high school, high school or more, unknown), body mass index (BMI) from baseline questionnaire, and BMI at Age 21 (both categorical: <18.5 kg/m^2, 18.5-<25.0 kg/m^2, 25-<30 kg/m^2, ≥30 kg/m^2), history of high blood pressure (yes, no), physical activity in Metabolic Equivalent Task-hours/week (quintiles), Alternative Healthy Eating Index diet scores (quintiles). Model 3 included adjustment of %AGA in models with SSDH-related factors as exposure and adjustment of racialized income ICE in models with %AGA. All models were fit using robust sandwich errors to account for census tract-level clustering.

© 2025 Iyer HS et al. *JAMA Network Open*.

eBox 1. Challenges and Recommendations for Appropriate Use of Genetic Ancestry in Health Disparities Research

Challenges

- Population descriptors used for genetic ancestry based on socially constructed continental and sociocultural labels may lead some to misattribute health effects of social causes to genetics.
- Due to sociopolitically derived referent labels, nomenclature used to define genetic ancestry is correlated with social constructs such as race and ethnicity and therefore correlated with non-genetic environmental causes of disparities.
- Naïve application of ancestry-based data and nomenclature may direct resources and treatment away from underrepresented racial and ethnic groups, thereby exacerbating health disparities.
- Socially-derived groupings may be more relevant than ancestrally-derived groupings to develop and implement interventions that address health disparities.

Recommendations

- When descent-related genetic variation is of interest in a study of racial disparities, genetic similarity measures are preferred because they avoid sociopolitical labels.
- Research on causal genetic variants to understand disease etiology may also involve metrics of genetic ancestry (e.g., by accounting for population stratification), but genetic ancestry should not be conflated with genetic etiology.
- Choice of measures (genetic, behavioral, health care access, environmental risk factors) should be guided by the research question and complete conceptual model for how disease arises.
- Studies of genetics and environment should be conducted in populations including diverse and underrepresented racial and ethnic groups to ensure generalizable and valid research results.

© 2025 Iyer HS et al. *JAMA Network Open*.

eTable 1. Population Descriptors used for Genetic Ancestry Clusters in the Multiethnic Cohort

Population Descriptor
**African Ancestry Continental Group**
**1000 Genomes**
Esan in Nigeria
Gambian in Western Division – Mandinka
Luhya in Webuye, Kenya
Mende in Sierra Leone
Yoruba in Ibadan, Nigeria
**Population Architecture using Genomics and Epidemiology**
AFRICA:ANGOLA
AFRICA:BOTSWANAORNAMIBIA
AFRICA:CENTRALAFRICANREPUBLIC
AFRICA:CONGO
AFRICA:KENYA
AFRICA:LESOTHO
AFRICA:NAMIBIA
AFRICA:NIGERIA
AFRICA:SENEGAL
**European Ancestry Continental Group**
**100 Genomes**
Utah Residents (CEPH) with Northern and Western European ancestry
British in England and Scotland
Iberian population in Spain
Toscani in Italia
**Population Architecture using Genomics and Epidemiology**
EUROPE:FRANCE
EUROPE:ITALY
EUROPE:ITALY-BERGAMO
EUROPE:ORKNEYISLANDS
**Ad Mixed American Ancestry Continental Group**
**Population Architecture using Genomics and Epidemiology**
AMERICA:BRAZIL
AMERICA:COLOMBIA
AMERICA:MEXICO
AMERICA:PERU
AMERICA:VENEZUELA
**East Asian Ancestry Continental Group**
**100 Genomes**
Chinese Dai in Xishuangbanna, China
Han Chinese in Beijing, China
Han Chinese South, China
Japanese in Tokyo, Japan
Kinh in Ho Chi Minh City, Vietnam
**Population Architecture using Genomics and Epidemiology**
EAST_ASIA:CAMBODIA
EAST_ASIA:CHINA
EAST_ASIA:JAPAN

© 2025 Iyer HS et al. *JAMA Network Open*.

eTable 2. Hazard Ratios[a] for Associations between Measures of Neighborhood Structural and Social Determinants with All-Cause Mortality Among Self-Identified Black Adults from the Multiethnic Cohort

	Continuous[e]	Quintile 1 (least disadvantaged)	Quintile 2	Quintile 3	Quintile 4	Quintile 5 (most disadvantaged)	P_{trend}
**Income ICE[f]**							
Cases/Person-years		363/17,494	704/30,451	694/24,116	1,387/50,705	2,356/81,696	
Mortality rate[g]		20.75	23.12	28.78	27.35	28.84	
Model 1(Minimal)[b]	1.22 (1.17, 1.28)	Ref	1.11 (0.96, 1.27)	1.39 (1.20, 1.60)	1.42 (1.26, 1.59)	1.54 (1.38, 1.72)	<.0001
Model 2 (Confounding)[c]	1.13 (1.08, 1.18)	Ref	1.06 (0.93, 1.22)	1.23 (1.08, 1.42)	1.26 (1.12, 1.42)	1.30 (1.16, 1.45)	<.0001
Model 3(Confounding + %AGA) [d]	1.12 (1.08, 1.17)	Ref	1.06 (0.93, 1.21)	1.22 (1.07, 1.4)	1.26 (1.12, 1.41)	1.29 (1.15, 1.44)	<.0001
**Race ICE[f]**							
Cases/Person-years		131/3,673	237/6,510	353/8,701	199/4,956	4,584/180,622	
Mortality rate[g]		35.67	36.41	40.57	40.15	25.38	
Model 1(Minimal)[b]	0.99 (0.96, 1.02)	Ref	1.03 (0.83, 1.27)	1.11 (0.91, 1.35)	1.24 (0.99, 1.54)	1.12 (0.94, 1.32)	0.33
Model 2 (Confounding)[c]	1.00 (0.97, 1.03)	Ref	0.87 (0.70, 1.08)	0.93 (0.77, 1.13)	0.98 (0.79, 1.22)	0.97 (0.82, 1.14)	0.34
Model 3(Confounding + %AGA) [d]	1.00 (0.97, 1.03)	Ref	0.86 (0.69, 1.07)	0.92 (0.76, 1.11)	0.97 (0.78, 1.21)	0.95 (0.81, 1.12)	0.38
**Racialized Income ICE[f]**							
Cases/Person-years		126/4,230	237/6,630	331/8,988	295/10,399	4,515/174,215	
Mortality rate[g]		29.79	35.75	36.83	28.37	25.92	
Model 1(Minimal)[b]	1.14 (1.09, 1.19)	Ref	1.27 (1.00, 1.62)	1.41 (1.14, 1.74)	1.55 (1.19, 2.02)	1.47 (1.22, 1.77)	0.0032
Model 2 (Confounding)[c]	1.08 (1.04, 1.13)	Ref	1.18 (0.93, 1.48)	1.26 (1.04, 1.55)	1.39 (1.09, 1.76)	1.30 (1.09, 1.55)	0.042
Model 3(Confounding + %AGA) [d]	1.08 (1.03, 1.12)	Ref	1.17 (0.93, 1.47)	1.25 (1.02, 1.53)	1.36 (1.07, 1.73)	1.28 (1.08, 1.53)	0.055
**nSES[f]**							
Cases/Person-years		234/8,765	711/33,555	945/36,122	1,868/66,109	1,746/59,912	
Mortality rate[g]		26.70	21.19	26.16	28.26	29.14	
Model 1(Minimal)[b]	1.25 (1.19, 1.31)	Ref	1.11 (0.95, 1.29)	1.38 (1.18, 1.60)	1.45 (1.27, 1.67)	1.67 (1.45, 1.91)	<.0001
Model 2 (Confounding)[c]	1.14 (1.09, 1.19)	Ref	1.07 (0.93, 1.24)	1.21 (1.05, 1.40)	1.28 (1.13, 1.46)	1.37 (1.20, 1.56)	<.0001
Model 3(Confounding + %AGA) [d]	1.14 (1.09, 1.19)	Ref	1.07 (0.93, 1.23)	1.2 (1.04, 1.39)	1.27 (1.11, 1.45)	1.36 (1.19, 1.55)	<.0001

Abbreviations: nSES=neighborhood socioeconomic status, ICE Inc=Income Index of Concentration at Extremes, [a]All models were adjusted for clustering at census tract level using robust sandwich errors. Models sequentially adjusted for [b]birth cohort and sex, [c]smoking status, marital status, educational attainment, Body Mass Index, Body Mass Index at Age 21, history of hypertension, history of diabetes, history of cardiovascular disease, history of cancer, physical activity, Alternative Healthy Eating Index, and [d] Percent African Genetic Ancestry. [e]Scaled to interquartile range for socioeconomic variables. [f]Cutpoints for quintiles were set based on census tract geographies in Los Angeles. Note: nSES and ICE measures were reverse coded, [g]Mortality rate per 1000 person-years.

© 2025 Iyer HS et al. *JAMA Network Open*.

eTable 3. Hazard Ratios[a] for Associations between Percent African Genetic Ancestry (%AGA) with All-Cause Mortality Among Self-Identified Black Adults from the Multiethnic Cohort

	Continuous[e]	Quintile 1 (Lowest %AGA)	Quintile 2	Quintile 3	Quintile 4	Quintile 5 (Highest %AGA)	P_{trend}
African Genetic Ancestry[f]							
Cases/Person-years		988/36,050	1,121/42,222	1,122/43,163	1,137/42,124	1,126/40,905	
Mortality rate[g]		27.41	26.55	25.99	26.99	27.53	
Model 1(Minimal)[b]	1.04 (1.03, 1.06)	Ref	1.12 (1.02, 1.22)	1.14 (1.05, 1.24)	1.22 (1.13, 1.33)	1.20 (1.11, 1.31)	<.0001
Model 2 (Minimal + nSES)[b]	1.03 (1.01, 1.05)	Ref	1.08 (0.99, 1.19)	1.09 (1.01, 1.19)	1.15 (1.06, 1.26)	1.14 (1.05, 1.25)	0.0005
Model 3 (Minimal + ICE Income)[b]	1.03 (1.01, 1.05)	Ref	1.09 (0.99, 1.20)	1.10 (1.01, 1.19)	1.17 (1.07, 1.27)	1.16 (1.06, 1.26)	0.0001
Model 4 (Minimal + Racialized Income ICE)[b]	1.04 (1.02, 1.06)	Ref	1.11 (1.01, 1.21)	1.13 (1.04, 1.23)	1.20 (1.11, 1.31)	1.19 (1.09, 1.29)	<.0001
Model 5 (Confounding)[c]	1.01 (0.99, 1.03)	Ref	1.06 (0.97, 1.17)	1.05 (0.96, 1.15)	1.11 (1.02, 1.21)	1.06 (0.97, 1.16)	0.085
Model 6 (Confounding + Racialized Income ICE)[d]	1.01 (0.99, 1.03)	Ref	1.06 (0.96, 1.16)	1.05 (0.96, 1.14)	1.10 (1.01, 1.20)	1.05 (0.97, 1.15)	0.11

Abbreviations: %AGA = African Genetic Ancestry, ICE Inc=Income Index of Concentration at Extremes, [a]Models adjusted for clustering at census tract level using robust sandwich errors. Models sequentially adjusted for [b]birth cohort and sex, [c]smoking status, marital status, educational attainment, Body Mass Index, Body Mass Index at Age 21, history of hypertension, history of diabetes, history of cardiovascular disease, history of cancer, physical activity, Alternative Healthy Eating Index, and [d]Racialized Income ICE. [e]Scaled to 10% AGA increase in African admixture (vs European). [f]Cutpoints for quintiles were set based on census tract geographies in Los Angeles.

© 2025 Iyer HS et al. *JAMA Network Open*.

eFigure 1. Spearman correlations for geospatial Social and Structural Determinants of Health (SSDH) with Percent African Genetic Ancestry (%AGA)

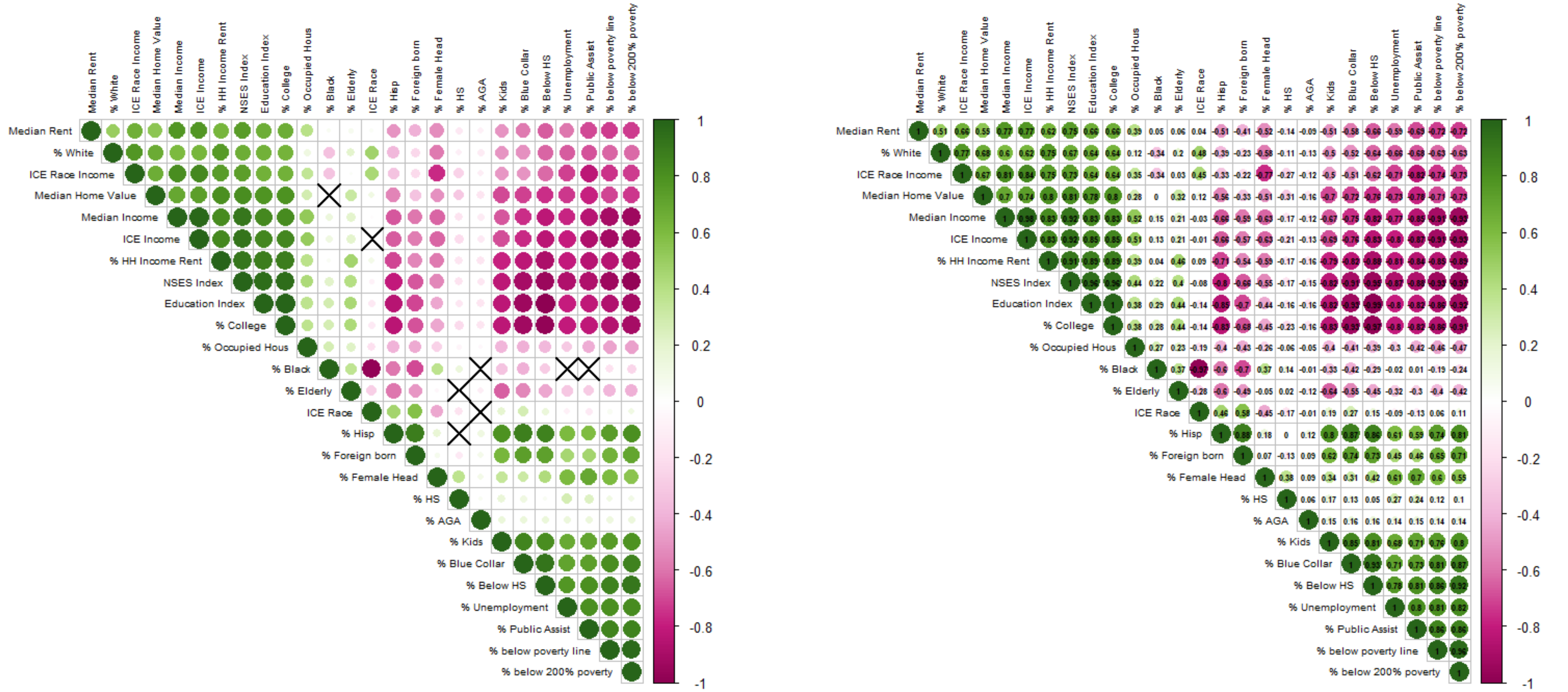

Legend: X = not statistically significant at two-sided α=0.05, Green = Positive correlation, Magenta=Negative correlation

© 2025 Iyer HS et al. *JAMA Network Open*.

eFigure 2. Kaplan-Meier Survival Curves for Associations of Percent African Genetic Ancestry (%AGA) and Structural and Social Determinants of Health (SSDH) with All-Cause Mortality among Self-Identified Black Participants from the Multiethnic Cohort using Los Angeles county-specific cutpoints

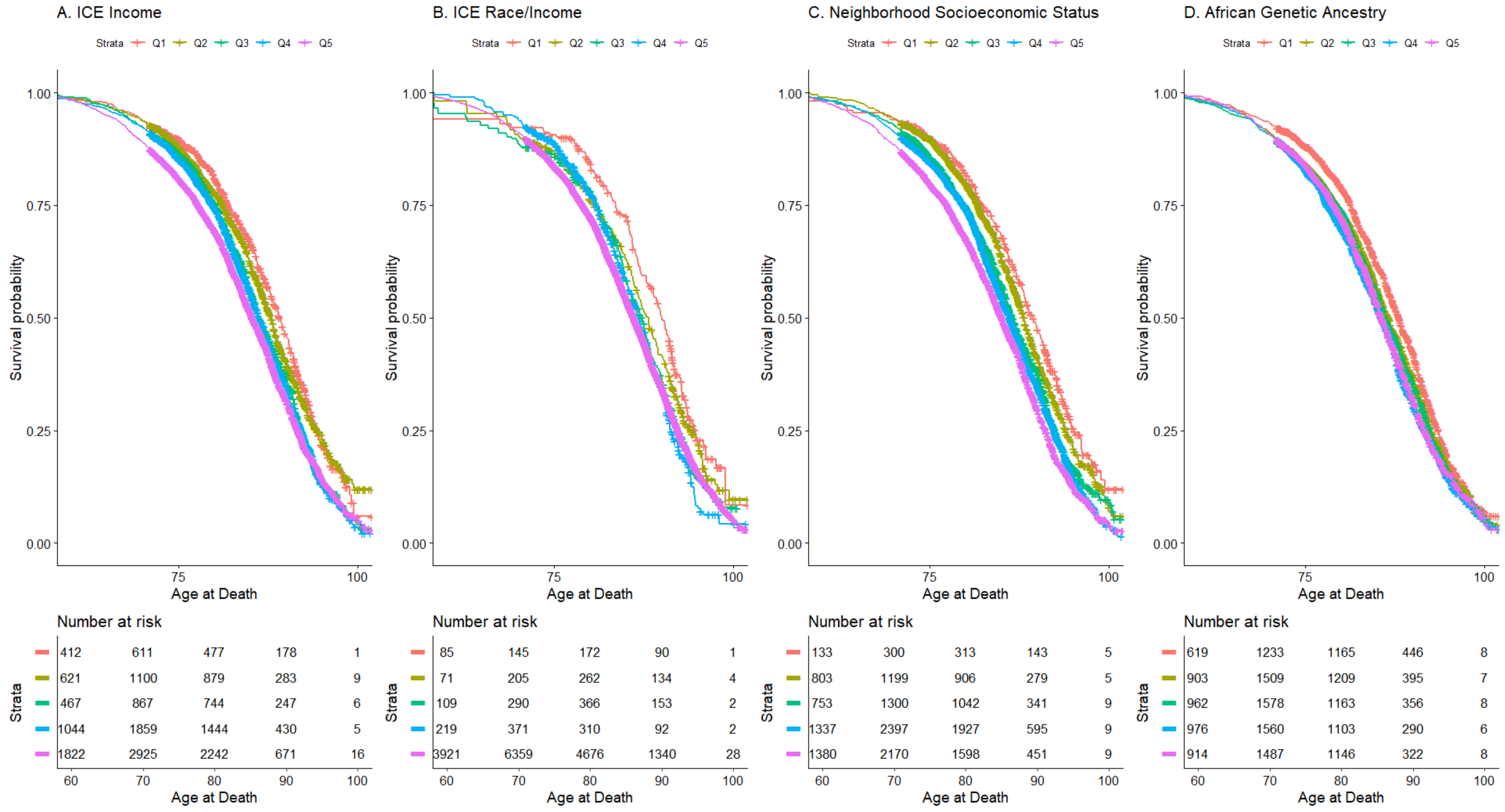

Legend: Log-rank p-values (A: <.0001, B: <.0001, C: <.0001, D: 0.40)

© 2025 Iyer HS et al. *JAMA Network Open*.
