# Supplementary material for: African Genetic Ancestry, Structural and Social Determinants of Health, and Mortality in Black Adults
Source: JAMA Netw Open. 2025 May 13;8(5):e2510016. doi: 10.1001/jamanetworkopen.2025.10016 (PMC12076178; doi:10.1001/jamanetworkopen.2025.10016)
Supplement: Supplement 2. — Data Sharing Statement [file jamanetwopen-e2510016-s002.pdf]

Data Sharing Statement

Iyer. African Genetic Ancestry, Structural and Social Determinants of Health, and Mortality in Black Adults. *JAMA Netw Open*. Published online May 13, 2025. doi:10.1001/jamanetworkopen.2025.10016

Data
Data available: No

Additional Information
Explanation for why data not available: The Multiethnic Cohort investigators and institutions affirm their intention to share the research data consistent with all relevant NIH resource/data sharing policies. Data requests should be submitted through MEC online data request system at https://www.uhcancercenter.org/for-researchers/mec-data-sharing .
